# Evaluating the Impact of a Social Games Club on International Students’ Mental Well-Being and Social Integration: A Mixed-Methods Study

**DOI:** 10.1192/bjo.2026.11228

**Published:** 2026-06-30

**Authors:** Favour Adanna Chimere, Victor Nwamara

**Affiliations:** NHS, Carlisle, United Kingdom

## Abstract

**Aims:**

This study aimed to evaluate the experiences and perspectives of international students participating in a games club, focusing on engagement, social interaction, and skill development across different cohorts and weeks.

Transitioning to university life presents significant challenges for international students, including social isolation, homesickness, and stress, which can affect mental well-being and academic performance. Structured social interventions offer potential benefits in supporting adaptation and promoting mental health.

**Methods:**

I coordinated a four-week games club at the University of South Wales for international MSc Public Health students. Weekly sessions included a variety of interactive games designed to foster social connection and participation. Participant feedback was collected via anonymous questionnaires after each session. Quantitative data were analysed descriptively, and qualitative responses underwent thematic analysis to identify patterns in engagement, enjoyment, and social integration.

**Results:**

Across the October and February cohorts, participants consistently reported high levels of enjoyment, with over 95% affirming positive experiences each week. Social interaction and engagement with multiple games were highest in the initial sessions and decreased in later weeks, particularly in the February cohort. Thematic analysis identified key themes including enhanced social well-being, gratitude for the initiative, and suggestions for improving inclusivity and activity variety. Participants reported skill development in teamwork, communication, and problem-solving.

**Conclusion:**

The games club effectively promoted social integration and mental well-being among international students. Observed variations in engagement highlight the importance of balancing novelty and familiarity, and designing interventions that sustain participation.These findings provide actionable insights for educators and public health practitioners developing targeted social interventions for diverse student populations. My role as facilitator and researcher enabled a nuanced understanding of participant experiences and informed recommendations for future iterations.

